# Metronidazole Induced Liver Injury: A Rare Immune Mediated Drug Reaction

**DOI:** 10.1155/2013/568193

**Published:** 2013-12-23

**Authors:** Dayakar Kancherla, Mahesh Gajendran, Priyanka Vallabhaneni, Kishore Vipperla

**Affiliations:** ^1^Division of General Internal Medicine, University of Pittsburgh, Pittsburgh, PA 15213, USA; ^2^Department of Internal Medicine, St. Luke's University Hospitals and Health Network, Bethlehem, PA 18015, USA

## Abstract

Drug induced liver injury (DILI) can result either from dose-dependent direct hepatotoxicity or from an unpredictable dose-independent idiosyncratic reaction. Incidence of idiosyncratic DILI is estimated to be approximately 10–15 per 100,000 patient years. Here we report an extremely rare case of metronidazole induced delayed immune-allergic hepatocellular liver injury masquerading as autoimmune hepatitis. A previously healthy 54-year-old Caucasian male, who was treated with metronidazole for *Clostridium difficile* associated diarrhea, presented 3 months later with right upper quadrant abdominal pain. Laboratory tests revealed total bilirubin level of 12.7 mg/dL, direct bilirubin of 7.2 mg/dL, alanine aminotransferase (ALT) of 973 IU/L, aspartate transaminase (AST) of 867 IU/L, alkaline phosphatase (AP) of 96 IU/L, and an INR of 1.9, suggestive of hepatocellular pattern of injury. A detailed workup for hepatitis revealed no other etiology. A clinical diagnosis of metronidazole induced liver injury was made. With a persistent rise in his bilirubin and transaminase levels, the patient was started on oral prednisone. At the 2-week posthospitalization follow-up visit, the patient reported a significant improvement in his overall sense of being well and liver functions tests trended down substantially (total bilirubin 7.2 mg/dL, ALT 420 IU/L, AST 276 IU/L, AP 183 IU/L, and INR 1.5).

## 1. Introduction

Reactive chemical metabolites formed during hepatic drug metabolism can incite hepatocellular damage from oxidative stress and mitochondrial dysfunction causing drug induced liver injury (DILI). DILI can result from either dose-dependent direct hepatotoxicity (e.g., acetaminophen toxicity) or from an unpredictable dose-independent idiosyncratic reaction. Genetic polymorphisms in the drug bioactivation and detoxification pathways along with host immunological factors are responsible for these rare and potentially fatal idiosyncratic DILI [1]. Of the several mechanisms proposed to elucidate the mechanism underlying immune-allergic idiosyncratic DILI, the “hapten hypothesis” is the most favored [2]. Drugs and/or their metabolites covalently bind to host proteins forming drug-protein adducts (i.e., haptens) that are processed by the antigen-presenting cells and trigger a T-cell mediated cytotoxicity or B-cell antibody response. Incidence of idiosyncratic DILI is estimated to be approximately 10–15 per 100,000 patient years [3]. About 1 in 7 cases of acute liver failure are related to an adverse drug reaction, making DILI the most common indication for liver transplantation in USA [4]. Antimicrobials are the most common class (~45%) of drugs responsible for DILI with amoxicillin-clavulanic acid being the single most common causative agent [5]. Advanced age, female sex, drug dose, and the extent of its hepatic drug metabolism are some of the identified risk factors for DILI [6]. A “probable” reaction to metronidazole presenting as a cholestatic pattern liver injury reaction within a few days after initiation and that resolved shortly after drug cessation has been reported earlier [7]. Here we report an extremely rare case of metronidazole induced delayed immune-allergic hepatocellular liver injury masquerading as autoimmune hepatitis that has not been previously reported to our knowledge.

## 2. Case Presentation

A previously healthy 54-year-old Caucasian male presented to our institution with worsening right upper quadrant abdominal pain and jaundice of 3-week duration. He denied alcohol or tobacco use, and his recent past medical history was significant only for a dental abscess treated with clindamycin followed by the development of *Clostridium difficile* associated diarrhea (CDAD) approximately 3 months prior to this admission. His CDAD was successfully treated then by a 2-week course of metronidazole, but the course was staggered due to the complaints of vague epigastric and right upper quadrant abdominal discomfort associated with nausea that were felt to be from drug “intolerance.” But his abdominal discomfort, anorexia, and nausea gradually worsened in the subsequent weeks though he denied having any skin rash, fever, vomiting, or change in bowel habits since his treatment with metronidazole. Three weeks ago, he started to notice darker urine, acholic stools, yellowish skin discoloration, and pruritus. “Abnormal liver function tests” noted on blood tests ordered by his family physician prompted an in-patient evaluation.

At admission, the patient was afebrile and had stable vital signs. Physical examination revealed a comfortable appearing well-built male who had remarkable icterus and jaundice without any other stigmata of chronic liver disease or cirrhosis such as spider nevi, clubbing, or muscle atrophy. Cardiovascular and respiratory system examination was normal. His abdomen was nondistended with normal bowel sounds, but was mildly tender in the right upper quadrant without signs of peritonitis, hepatosplenomegaly, or ascites. His mental status was intact and did not exhibit asterixis. Initial blood tests (normal values range in parenthesis) revealed a total bilirubin level of 12.7 (0.3–1.5) mg/dL, direct bilirubin of 7.2 (0.1–0.5) mg/dL, alanine aminotransferase (ALT) of 973 (17–63) IU/L, aspartate transaminase (AST) of 867 (15–41) IU/L, alkaline phosphatase (AP) of 96 (38–126) IU/L, and an INR of 1.9 (0.8–1.2) clearly suggestive of mild hepatic failure with a hepatocellular pattern of injury. His complete blood count and renal function tests were within normal limits. A right upper quadrant abdominal Doppler ultrasonography revealed normal hepatic texture and patent hepatic and portal veins. A magnetic resonance cholangiopancreatography (MRCP) scan was remarkable for moderate periportal edema but otherwise normal hepatic morphology and without any obstruction within the biliary tract. However during the gastroenterologist's personal review of the MRCP images, a subtle filling defect was suspected in the proximal CBD prompting an endoscopic retrograde cholangiography (ERCP) which did not reveal any obstructive process or mass.

**Figure 1:**
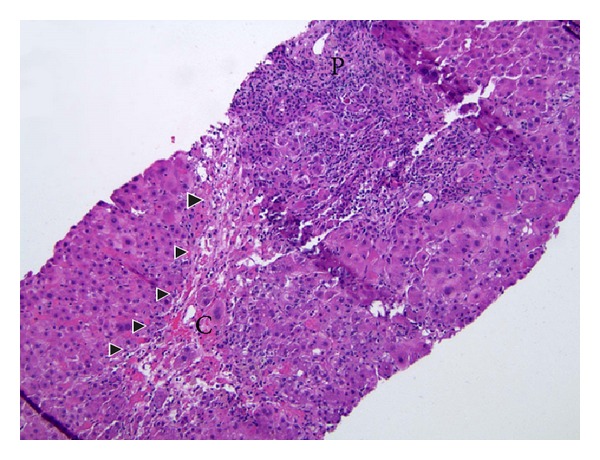
The liver biopsy showed severe portal and lobular hepatitis with perivenular and bridging necrosis (arrow heads) (H&E stain, original magnification 100x). P: portal tracts, C: central vein.

**Figure 2:**
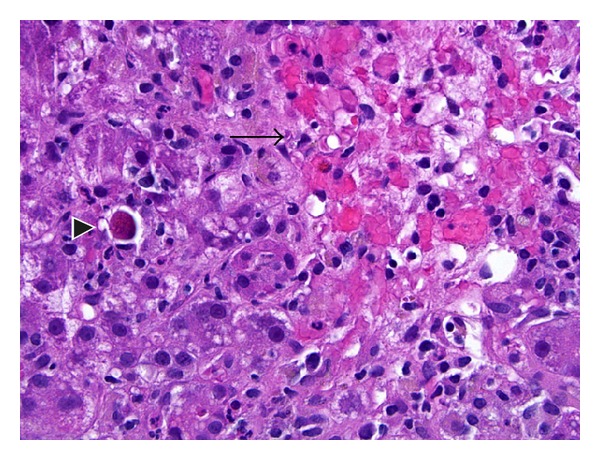
High-magnification picture of the lobule showing an area of centrizonal confluent necrosis (arrow) and adjacent viable parenchyma. Scattered mononuclear inflammatory cells and an apoptotic hepatocyte are present in the viable parenchyma (H&E stain, original magnification 400x).

**Figure 3:**
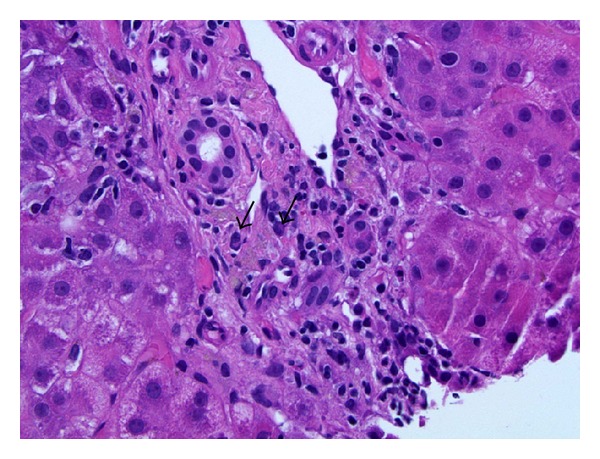
Infiltrating cells in the portal tracts were predominantly lymphocytes admixed with fewer neutrophils, plasma cells (arrows), and rare eosinophils (H&E stain, original magnification 400x).

During a 4-day hospitalization, a comprehensive laboratory workup was performed to rule out acute and chronic liver diseases. Drug toxicity (undetectable serum acetaminophen levels), viral hepatitis (HIV, herpes simplex virus, hepatitides A, B, C, E antibodies, Epstein Barr virus, and cytomegalovirus PCR), autoimmune hepatitis (anti-nuclear antibody, anti-neutrophil cytoplasmic antibody, anti-M2 mitochondrial antibody, anti-smooth muscle antibody, and quantitative immunoglobulin levels), and metabolic liver disease (serum ceruloplasmin and alpha-1 antitrypsin levels) tests were unremarkable. Progressively worsening hepatic transaminase levels prompted a liver biopsy for a definitive diagnosis. An ultrasound guided needle biopsy specimen from right hepatic lobe showed severe acute to subacute predominantly portal and lobular hepatitis with confluent perivenular and bridging necrosis strongly suggestive of an immunoallergic etiology related to either a drug reaction or autoimmune hepatitis (Figures 1, 2, and 3). His age, sex, and clinical presentation were not typical of autoimmune hepatitis, and, moreover, the autoimmune serological workup was not corroborative, making a drug reaction to metronidazole the most likely etiology.

With a persistent rise in his bilirubin and transaminase levels, the patient was started on oral steroids with prednisone prescribed at a dose of 40 mg/day to control hepatic inflammation contributing to the hepatocellular injury. He was clinically stable and symptomatically comfortable throughout the hospitalization without developing signs or symptoms of fulminant hepatic failure. He was discharged home with recommendation to use a 4-week course of steroids with close followup of his liver function tests. At the 2-week posthospitalization followup visit, the patient reported a significant improvement in his overall sense of being well and liver functions tests trended down substantially (total bilirubin level of 7.2 mg/dL, ALT of 420 IU/L, AST of 276 IU/L, AP of 183 IU/L, and an INR of 1.5). He was recommended to continue using the steroid for a total of 4 weeks with subsequent gradual tapering of the steroid dose with close monitoring of his liver function test. Excellent response to prednisone and absence of a preexisting underlying liver disease predicts good prognosis, but only time would determine if he would have any long-lasting sequelae of DILI.

## 3. Discussion

Immunoallergic DILI is a form of DILI that typically presents 1–3 months after exposure to the drug, the time that typically is taken for manifestation of a full-blown delayed immune reaction. DILI can present as a wide spectrum ranging from asymptomatic elevation of liver enzymes to fulminant hepatic failure and often mimics other acute or chronic liver diseases. The abnormal liver enzymes can be categorized into cholestatic, hepatocellular, or mixed injury patterns. It is a diagnosis of exclusion that is established by a strong clinical suspicion and a comprehensive workup to rule out other competing etiologies. Diagnostic algorithms such as Roussel Uclaf Causality Assessment Model have been proposed to assist in ascertaining the probability of DILI but are not robust for general clinical application. Histopathological findings of DILI are nonspecific too but can be valuable in narrowing the differential diagnoses.

Treatment measures mainly involve prompt cessation of the offending drug and supportive care. N-Acetyl cysteine has been used in some cases and was shown to improve transplant free survival, though mortality reduction benefit was not observed [8]. Treatment with steroids has been of unproven benefit in most hepatotoxic drug reactions but offers a potential therapeutic role when DILI is secondary to a hypersensitivity reaction and has a severe clinical course or worsening liver function tests with conservative management, identical to our case [9]. Majority of patients surviving DILI recover completely, but ~6% persist to have chronic liver disease (chronic DILI), and only 1% develop “cryptogenic” cirrhosis [10]. Interestingly, 22% of patients with chronic DILI at presentation developed autoimmune hepatitis eventually in one registry, suggesting the possibility of drug induced increase in susceptibility to developing autoimmune hepatitis.

The wide spread antibiotic usage in the practice of modern medicine has been responsible for a surge in potentially life threatening drug reactions. Drug reactions that develop immediately after initiation of inciting agent are easier to recognize and resolve with cessation of the drug. But it is quite challenging to recognize the rare immune mediated delayed reactions. Albeit unavoidable at the first instance, it is paramount to identify such idiosyncratic DILI to prevent recurrent reactions that are likely to be more severe and rapid in recurrence on rechallenge. Antibiotics have to be used judiciously bearing in mind the risk of DILI even for a widely used drug such as metronidazole, which is generally considered to be quite a safer drug for short-term use. Also, potentially serious adverse effects such as DILI have to be considered in the differential diagnosis in the work up for jaundice.
